# Correlation Between Dissolution Profiles of Salt-Form Drugs in Biorelevant Bicarbonate Buffer and Oral Drug Absorption: Importance of Dose/ Fluid Volume Ratio

**DOI:** 10.1007/s11095-025-03854-y

**Published:** 2025-04-04

**Authors:** Yuki Tarumi, Yuji Higashiguchi, Kiyohiko Sugano

**Affiliations:** Molecular Pharmaceutics Lab, College of Pharmaceutical Sciences, Ritsumeikan University, 1-1-1, Noji-Higashi, Kusatsu, Shiga 525-8577 Japan

**Keywords:** bicarbonate, dissolution test, prediction, salt form

## Abstract

**Purpose:**

The purpose of this study was to investigate the correlation between the dissolution profiles of salt-form drugs in biorelevant bicarbonate buffer and oral drug absorption.

**Methods:**

Ciprofloxacin HCl (CPFX HCl), garenoxacin mesylate (GRNX MS), tosufloxacin tosylate (TFLX TS), levofloxacin free-form (LVFX FF), and sitafloxacin free-form (STFX FF) were employed as model drugs. Bicarbonate buffer fasted state simulated intestinal fluid (BCB-FaSSIF) was used as a biorelevant dissolution medium (pH 6.5, BCB 10 mM (floating lid method), taurocholic acid (3 mM) and lecithin (0.75 mM)). The fraction of a dose absorbed in humans (*Fa*) was predicted by a simple theoretical framework for oral drug absorption using equilibrium solubility at pH 6.5 (*S*_*eq,pH6.5*_) or average dissolved drug concentration in the dissolution tests (*C*_*dissolv,AV*_).

**Results:**

*Fa* was adequately predicted using *S*_*eq,pH6.5*_ for LVFX FF and STFX FF, however, underpredicted for CPFX HCl (tenfold), GRNX MS (twofold), and TFLX TS (sevenfold). When compendial *Dose/FV* was used for the dissolution test of CPFX HCl, bulk pH (pH_bulk_) remained unchanged and *C*_*dissolv,AV*_ ≈ *S*_*eq,pH6.5*_, resulting in a tenfold underprediction of *Fa*. Using clinical *Dose/FV*, pH_bulk_ was decreased, *C*_*dissolv,AV*_ was increased, resulting in adequate *Fa* prediction. Similarly, for GRNX MS and TFLX TS, *Fa* predictability was improved using *C*_*dissolv,AV*_ at clinical *Dose/FV*. In these conditions, *C*_*dissolv,AV*_ > *S*_*eq,pH6.5*_ due to decreased pH_bulk_ below the first p*K*_*a*_ of the drugs.

**Conclusion:**

The use of clinical *Dose/FV* was important for improving the correlation between the biorelevant dissolution profiles and *Fa* for salt-form drugs.

**Supplementary Information:**

The online version contains supplementary material available at 10.1007/s11095-025-03854-y.

## Introduction

The rate and extent of oral drug absorption are determined by the dissolution and permeation profiles of a drug in the gastrointestinal (GI) tract [1, 2]. In drug discovery and development, it is important to employ dissolution tests that correlate with clinical oral drug absorption [3]. In the case of free-form drug substances, previous systematic studies have shown that the fraction of a dose absorbed (*Fa*) in humans is predictable with practical accuracy based on the intrinsic physicochemical properties such as the intrinsic solubility (*S*_*0*_) and the acid–base dissociation constant (p*K*_*a*_) [4–6]. However, *Fa* prediction has been challenging for salt-form drugs because supersaturation/ precipitation of a free-form drug that occurs after the dissolution of a salt-form drug is difficult to predict from the intrinsic physicochemical properties of a drug. Therefore, *Fa* prediction based on the correlation between the dissolution/ supersaturation/ precipitation profiles in dissolution tests (hereinafter referred to as dissolution profile) and clinical oral drug absorption would be a practical approach for salt-form drugs [7].

Dissolution tests have been used to predict the dissolution profile of a drug in the GI tract [8, 9]. In general, the closer the dissolution test conditions are to the *in vivo* GI tract (bio-relevant), the more accurate the oral absorption prediction (*in vivo* predictive) [10–12]. The physiological buffer species in the intestinal fluid is bicarbonate buffer (BCB) [13, 14]. Nevertheless, phosphate buffer (PPB) has been widely used for dissolution tests in practice [15]. However, the dissolution profiles in BCB and PPB show significant differences for many drugs [16, 17].

PPB and BCB differ in the rates and extents of pH neutralization, even when their pH and buffer capacity (β) values are set equal. Due to the slow hydration/ dehydration reaction of BCB (CO_2_ + H_2_O ⇄ H_2_CO_3_), BCB and PPB differently affect the surface pH (pH_surface_) of either dissolving [18, 19] or precipitating [20] drug particles. In addition, BCB and PPB differ in their ability to maintain the bulk phase pH (pH_bulk_) even when their β values are set equal [21, 22]. β is defined as the derivative of pH and the molar concentration of added acid or base (*n*, mol/L) (β = d*n*/dpH). The change of the bulk phase pH (∆pH_bulk_) is determined by the integral of 1/β for *n*. β becomes maximum where pH = p*K*_*a*_ (BCB: p*K*_*a*_ = 6.05, PPB: p*K*_*a*_ = 6.69, 37˚C, ionic strength (*I*) = 0.15 M) [23]. Therefore, when started from pH 6.5, BCB maintains the bulk phase pH value more efficiently than PPB for acidification, but the opposite for alkalization [24]. The typical buffer concentration in compendial media is much higher than that *in vivo* [25]. In addition, some weakly basic drugs form an insoluble salt with phosphate ions [26, 27].

The dissolution profiles of salt-form drugs markedly differ in PPB and BCB [17, 24, 28]. The initial dissolution rate of salt-form drug particles is usually rapid. However, a poorly soluble free form can rapidly precipitate on the surface of salt-form particles and inhibit the dissolution process [28–30]. In addition, the precipitation of a free-form solid occurs in the bulk phase [20]. Precipitation is a sequential process of nucleation and particle growth [31–33]. After the dissolution of a salt form drug, the drug molecules are in equilibriums of charged and uncharged species in an aqueous solution (charge: *z*,* z* = 0 for un-ionizable molecules or net *z* = ± 0 for zwitterions). In the neutral pH region, uncharged species show the least solubility in most cases. The fraction of uncharged species (*f*_*0*_) depends on the p*K*_*a*_(s) of a drug and the pH of a solution (pH_surface_ or pH_bulk_). Precipitation of the free-form solid can occur either on the surface of salt-form particles or in the bulk phase where the concentration of uncharged molecular species (*C*_*0*_) exceeds *S*_*0*_ of the free-form solid [29, 34, 35]. The precipitation induction time steeply depends on the supersaturation ratio (*SR*_*0*_ = *C*_*0*_*/S*_*0*_) [36]. Both particle surface and bulk phase precipitations can be affected differently by BCB and PPB [20, 28].

In addition to buffer conditions, the dose/fluid volume ratio (*Dose/FV*) is an important factor for the dissolution test of salt-form drugs [24, 37]. A non-sink condition is required for a dissolution test to assess supersaturation/ precipitation phenomena. In compendial dissolution tests, the fluid volume of 500 to 900 mL is often used to ensure a sink condition. On the other hand, the small intestinal fluid volume (*V*_*SI*_) is about 100 mL or less in humans [37–39]. Therefore, drug dissolution tends to proceed under a non-sink condition *in vivo*, especially when the permeation clearance of a drug is low to moderate. In addition, *Dose/FV* affects the change in pH_bulk_ during a dissolution test when using biorelevant BCB buffer [24]. In the previous study, it was qualitatively suggested that the use of clinically relevant *Dose/FV* is important for predicting the oral absorption of various salt-form drugs [24]. However, the quantitative *Fa* predictability has not been investigated.

**Fig. 1:**
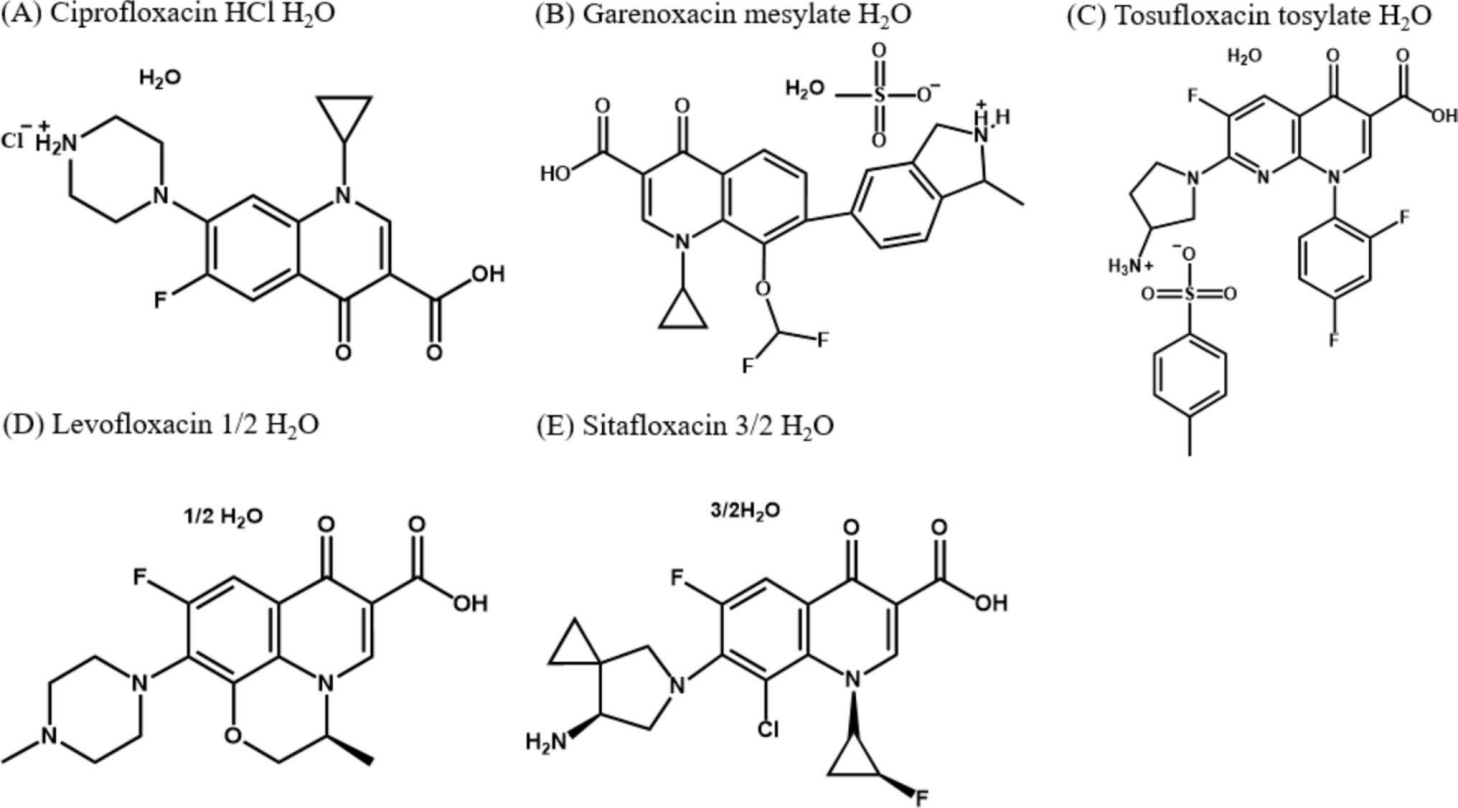
Chemical structures of fluoroquinolones.

The purpose of the present study was to evaluate the correlation between the dissolution profiles in biorelevant bicarbonate buffer at clinically relevant *Dose/FV* and the fraction of a dose absorbed in humans for salt-form drugs. Five fluoroquinolones were selected as model drugs (ciprofloxacin hydrochloride (CPFX HCl), garenoxacin mesylate (GRNX MS), tosufloxacin tosylate (TFLX TS), levofloxacin free form (LVFX FF), and sitafloxacin free form (STFX FF)) (Fig. 1, Tables I and II). The free-form drugs (LVFX FF and STFX FF) are employed for comparison. The model drugs are selected from one chemical series, fluoroquinolone, to increase the reliability of the permeability estimation based on the physicochemical properties (see discussion). BCB and BCB-based fasted state simulated intestinal fluid (BCB-FaSSIF) were used as a practical biorelevant simulated intestinal fluid (pH 6.5, 10 mM BCB, with/without 3 mM taurocholic acid (TC)/ 0.75 mM egg lecithin (EL), ionic strength (*I*) = 0.14 (adjusted by NaCl))[28, 40]. Dissolution tests were performed at *Dose/FV* that reflect the clinical dose and the intestinal fluid volume. For CPFX HCl, a modified version of USP phosphate buffer (pH 6.5, 50 mM) (USP_pH6.5_) and compendial *Dose/FV* were additionally used for comparison. To minimize the effect of the gastric dissolution process, clinical *Fa* data in humans with a hypo-acid gastric condition (coadministration of an H2 blocker or a proton pump inhibitor) were collected from the literature. *Fa* was predicted from the dissolution profiles by a simple and transparent theoretical framework for oral drug absorption (the GUT framework)[41], and compared with the clinical *Fa* data.

**Table I:** Physicochemical Properties of Fluoroquinolones

Drug	MW ^a^	p*K*_*a*_^b^	log ${D}_{oct}$ ^g^	*S*_*eq*_ (ug/mL) ^j^		Ref
				Blank FaSSIF	FaSSIF	
CPFX	331	6.15 (A), 8.63 (B) ^c^	−1.38 ^h^	160 ± 4	167 ± 1	[42–44]
GRNX	426	5.6 (A), 9.4 (B) ^d^	−0.43 ^i^	183 ± 1	202 ± 1	[45]
TFLX	404	5.8 (A), 8.7 (B) ^d^	−0.14 ^h^	2.5 ± 0.1	4.1 ± 0.2	[46]
LVFX	361	5.89 (A), 8.59 (B) ^e^	−0.56 ^h^	- ^k^	- ^k^	[23, 47]
STFX	410	5.7 (A), 9.1 (B) ^f^	−0.73 ^i^	178 ± 1	181 ± 4	[48, 49]

a As of free-form

b A: acid, B: base

c 24℃, *I* = 0.16 M

d No information on temperature and *I*

e 25℃, *I* = 0.15 M

f 25℃, No information on *I*

g pH 6.5

h Ref. [50]

i Measured in this study.

J Equilibrium solubility in the PPB-based fasted state simulated intestinal fluid (pH 6.5) with or without bile micelles. Mean ± SD (*N* = 3)

k The intrinsic solubility of levofloxacin was reported to be > 30 mg/mL [51]

**Table II:** Formulations Used in This Study

Drug	Solid form in the formulation	Formulations	Manufacturer
CPFX	HCl salt, monohydrate	Ciprofloxacin hydrochloride tablets 200 mg	Nichi-Iko Pharmaceutical Co., Ltd
GRNX	Mesylate salt, monohydrate	Geninax mesylate tablets 200 mg	Taisho Pharmaceutical Co., Ltd
TFLX	Tosylate salt, monohydrate	Tosufloxacin tosylate tablets 150 mg 「SW」	Sawai Pharmaceutical Co., Ltd
LVFX	Free form, hemihydrate	Levofloxacin tablets 250 mg 「DSEP」	Daiichi Sankyo Espha Co., Ltd
STFX	Free form, sesquihydrate	Sitafloxacin tablets 50 mg	Sawai Pharmaceutical Co., Ltd

## Materials and Methods

### Materials

Ciprofloxacin hydrochloride hydrate (CPFX HCl) and levofloxacin (LVFX FF) were purchased from Tokyo Chemical Industry (Tokyo, Japan). Tosufloxacin tosylate hydrate (TFLX TS), sodium hydrogen carbonate, sodium chloride, sodium dihydrogen phosphate dihydrate, 1-octanol, 6 mol/L hydrochloride, 8 mol/L sodium hydroxide solution, and taurocholic acid (TC) were purchased from Wako Pure Chemical Industries, Ltd. (Osaka, Japan). Egg yolk lecithin (EL) was purchased from Kewpie Corporation (Tokyo, Japan). The drug products were purchased from the Japanese market. The manufacturers of the drug products are summarized in Table II.

Garenoxacin free form hydrate (GRFX FF) and sitafloxacin free form hydrate (STFX FF) were extracted from the tablet. TFLX TS was converted to the corresponding free-form hydrate (TFLX FF) by adding an equimolar amount of 1 N NaOH. Similarly, CPFX HCl was converted to its free-form hydrate (CPFX FF). Because this procedure yielded a mixture of CPFX FF hydrate Form II and Form III [52], it was converted to CPFX FF anhydrate (CPFX FF AH) by heating at 120℃ for 15 min. To prepare the reference material of CPFX FF hydrate Form III, CPFX FF AH was suspended in distilled water for 3 days at 37℃.

The hydrate number of GRNX FF hydrate and TFLX FF hydrate were determined by thermogravimetric analysis (TGA) (Supplemental information (SI) Figure S1) (GRNX FF 3H_2_O and STFX FF 1.5H_2_O, respectively). The solid forms of CPFX FF AH, CPFX FF hydrate Form III, and STFX FF 1.5H_2_O were identified by powder X-ray diffraction (PXRD) data in the literature (SI Figure S1, Figure S4 A (reference material), and Figure S4 D (reference material), respectively) [52, 53]. CPFX FF AH was used for solubility measurement because CPFX FF hydrate Form III was difficult to weigh due to its rapid conversion to a mixture of Form II and Form III at ambient humidity.

## Methods

### Octanol–Water Distribution Coefficient

The octanol-buffer distribution coefficient at pH 6.5 (log *D*_*oct, pH6.5*_) was determined by a shake-flask method. The octanol and buffer phases (50 mM sodium-phosphate buffer, pH 6.5) were mutually pre-saturated before use. A drug buffer solution (0.05 mM, 0.5 mL) and octanol (2.5 mL) were added to a 15 mL tube. The sample was vigorously shaken for 90 min at room temperature (25 ± 2℃). The drug concentration in the aqueous phase was measured by UV absorbance. The detection wavelength, the concentration range, and the determination coefficient (r^2^) of standard curves are summarized in SI Table S1. Log *D*_*oct, pH6.5*_ was measured in triplicate. *D*_*oct, pH6.5*_ was calculated as

1 $${D}_{oct, pH 6.5}=\frac{{C}_{aq,ini}-{C}_{aq,eq}}{{C}_{aq,eq}}\frac{{V}_{aq}}{{V}_{oct}}$$

where *C*_*aq,ini,*_ and *C*_*aq,eq*_ are the initial and equilibrium drug concentrations in the aqueous phase, respectively, and *V*_*aq*_ and *V*_*oct*_ are the volume of aqueous and octanol phases, respectively.

## Equilibrium Solubility Measurement

The PPB-based fasted state simulated intestinal fluid (PPB-FaSSIF) (pH6.5, 28.7 mM phosphate, 3 mM TC, 0.75 mM EL) or that without bile micelle (blank PPB-FaSSIF) (10 mL) was added to a 15 mL vial. An excess amount of each free-form drug (CPFX FF anhydrate: 20 mg, TFLX FF 2H_2_O: 10 mg, GRNX FF 3H_2_O: 15 mg, and STFX FF 1.5H_2_O: 15 mg) was added to a vial and capped tightly. The vial was placed horizontally on a horizontal shaker, and strongly agitated (90 rpm, oscillation width: 2.5 cm) for 72 h at 37℃. A sample solution (1 mL) was collected and immediately filtered (0.22 µm hydrophilic PVDF filter, φ = 4.0 mm, Millipore Corporation, Billerica, MA, USA). The first few drops of the filtrate were discarded to avoid filter adsorption. The filtrate was diluted with an appropriate medium, and the drug concentration was measured by UV absorbance (Table II) (SH1000lab, Hitachi High-Technologies Corporation, Tokyo, Japan). The final pH_bulk_ was measured using a Gel-filled pH sensor cartridge 300-P–C (HORIBA Advanced Techno, Co., Ltd., Kyoto, Japan). The final pH_bulk_, the detection wavelength, the concentration range, and the determination coefficient (r^2^) of standard curves are summarized in SI Table S2. The equilibrium solubility (*S*_*eq*_) was measured in triplicate.

The residual solid was collected by vacuum filtration and the solid form was determined by powder X-ray diffraction (PXRD, Rigaku Ultima IV, Rigaku Corporation, Tokyo, Japan) and differential scanning calorimetry (DSC, DSC60 plus, Shimadzu Corporation Kyoto, Japan). Before PXRD analysis, samples were gently ground with a mortar and pestle to avoid the orientation of crystals. A zero-diffraction plate was used as a sample holder. PXRD data were measured from 5° to 35° (2θ) (scanning speed: 10°/min; step size: 0.02°; Cu Kα radiation (40 mA, 40 kV)). For CPFX, samples were measured wet with water to avoid conversion to hydrate Form II. In the DSC measurement, samples were placed in an aluminum pan (non-sealed) and measured under nitrogen gas at 10℃/min.

## Dissolution Test

A paddle dissolution apparatus with a mini-vessel or a compendial vessel was used for the dissolution test (NTR-6200A, Toyama Sangyo Co., Ltd., Osaka, Japan). The fluid volume was 50 mL (mini vessel) or 900 mL (compendial vessel). BCB was prepared as follows. A NaCl solution (0.168 mol/L) was added to each vessel (mini-vessel (39.2 mL)/ compendial-vessel (705.6 mL)). The temperature was maintained at 37℃. The paddle rotation speed was set to 50 rpm. A NaHCO_3_ solution (51 mmol/L) was added to the vessels (mini-vessel (9.8 mL)/ compendial-vessel (176.4 mL)). An HCl solution (0.165 mol/L) was added to adjust the pH_bulk_ to 6.5 (mini-vessel (1.0 mL)/ compendial-vessel (18 mL)). The final buffer conditions were BCB = 10 mmol/L, β = 4.4 mM/pH unit, and *I* = 0.14 M. The β value was calculated by the van Slyke equation [54] assuming the activity coefficient is 1. For BCB-FaSSIF, a 100 × concentrated TC/EL solution in distilled water was added to give the final bile micelle concentration of TC: 3 mM and EL: 0.75 mM. The USP compendial phosphate buffer was modified to pH 6.5 (USP_pH6.5_). The floating lid (SI Figure S3) was used for all BCB, BCB-FaSSIF, and USP_pH6.5_ to align the experimental condition [55]. The *Dose/FV* value was set to reflect the conventional dissolution test or the clinical *Dose/FV*. A portion of a tablet, gently crushed in a mortar and pestle, was added to each vessel. At specified time intervals, a small volume of samples (500 µL) was withdrawn and immediately filtered (0.22 µm hydrophilic PVDF filter, φ = 4.0 mm, Millipore Corporation, Billerica, MA, USA). The first few droplets were discarded to avoid filter adsorption. The filtrate was diluted with an appropriate medium, and the drug concentration (*C*_*dissolv*_) was measured by UV absorbance (UV 1850, Shimazu Corporation, Kyoto, Japan, or SH-9500lab, CORONA ELECTRIC, Ibaraki, Japan). The detection wavelength, the concentration range, the number of data points, and the determination coefficient (r^2^) of standard curves are summarized in SI Table S3. The absence of UV interference from the excipients was confirmed by comparing the UV spectrum of a pure API and its drug product. The bulk phase pH value at 4 h (pH_bulk,4h_) was measured as described above. The dissolution test was performed in triplicate.

The area under the dissolution curve (AUDC) was calculated from the *C*_*dissolv*_—time profile by the trapezoidal method (0 to 3.5 h). The AUDC of 150 to 210 min was calculated by taking the average of the 150- and 240-min values.

## *Fa* Prediction from *In Vitro* Dissolution Profile

The *Fa* value was predicted from the dissolution profiles using the GUT framework [41]. The average of *C*_*dissolv*_ (*C*_*dissolv,av*_) was calculated as

2 $${C}_{dissolv,AV}=\frac{AUDC}{{T}_{SI}}$$

where *T*_*SI*_ is the small intestinal transit time (*T*_*SI*_ = 3.5 h). The dose number (*Do*) [1] was calculated as

3 $$Do=\frac{Dose}{{C}_{dissolv,AV}\times {V}_{SI}}$$

where*V*_*SI*_ is the effective intestinal fluid volume (*V*_*SI*_ = 130 mL) [4, 56]. The permeation number (*Pn*) was calculated as [57]

4 $$Pn={k}_{perm}{T}_{SI}=\frac{2DF}{{R}_{GI}}{P}_{eff}{T}_{SI}$$

where *k*_*perm*_ is the permeation rate coefficient, *DF* is the degree of flatness of the small intestine (*DF* = 1.7), *R*_*GI*_ is the radius of the small intestine (*R*_*GI*_ = 1.5 cm), and *P*_*eff*_ is the effective intestinal membrane permeability. *P*_*eff*_ was calculated as

5 $${P}_{eff}=\frac{PE}{\frac{1}{{P}_{UWL}}+\frac{1}{{P{\prime}}_{ep}}}=\frac{PE}{\frac{1}{{P}_{UWL}}+\frac{1}{VE{f}_{u}{P}_{ep}}}$$

6 $${P}_{UWL}=\frac{{D}_{eff}}{{h}_{eff}}+{P}_{wc}=\frac{{{f}_{u}D}_{mono}+\left(1-{f}_{u}\right){D}_{bm}}{{h}_{eff}}+{P}_{wc}$$

where *PE* and *VE* represent the expansion of surface area by plicae and villi structures), respectively (*PE* = 3 and *VE* = 10), *P*_*ep*_ is the intestinal epithelial membrane permeability, *f*_*u*_ is the free (unbound) fraction in bile micelle media, and *P*_*wc*_ is permeation by water conveyance (0.4 × 10^–4^ cm/s). *D*_*mono*_ and *D*_*bm*_ are the diffusion coefficients of monomer and bile micelle-bound drug species (*D*_*bm*_ = 3.8 × 10^–7^ cm^2^/s), and *h*_*eff*_ is the thickness of the unstirred water layer (*h*_*eff*_ = 332 μm). The *P*_*ep*_ value of CPFX was assumed to be the same as the apparent permeability value in the Caco-2 assay (2.9 × 10^–6^ cm/s) reported in the literature [42]. The *P*_*ep*_ values of the other fluoroquinolones were estimated based on this value by assuming being proportional to *D*_*oct, pH6.5*_. ${f}_{u}$ was calculated as the ratio of equilibrium solubility in blank PPB-FaSSIF to that in PPB-FaSSIF. *D*_*mono*_ in water at 37˚C was calculated as *D*_*mono*_ (cm^2^/s) = 9.9 × 10^–5^ × MW^−0.453^ [23]. *Fa* was predicted by the *Fa* equation (Eq. 6) [58], assuming rapid dissolution (dissolution number *Dn* > > *Pn*/*Do*).

7 $$Fa=1-\text{exp}(-\frac{1}{\frac{1}{Dn}+\frac{Do}{Pn}})\approx 1-\text{exp}(-\frac{Pn}{Do})$$

When *Do* < 1, set *Do* = 1.

## Clinical Fa Data

The clinical *Fa* values were estimated from the pharmacokinetic data in the literature.

### Ciprofloxacin HCl

When 750 mg of CPFX HCl was orally administered in the fasted state 2 h after ranitidine administration, AUC was 12.6 μg h/ mL [59–61]. After the i.v. administration of 50 to 400 mg, the average AUC was 20.8 μg h/ mL (normalized to 750 mg). From these data, the absolute bioavailability (*F*) was calculated to be 0.60. As CPFX was excreted mostly unmetabolized, *Fa* was assumed to be the same as *F* (*Fa*_*obs*_ = 0.60).

### Garenoxacin Mesylate

The absolute bioavailability (*F*) of GRNX mesylate (MS) after oral administration of 600 mg dose was reported to be 0.92 [45]. The AUC was not affected by the co-administration of omeprazole [45]. As GRNX was little metabolized, *Fa* was assumed to be the same as *F* (*Fa*_*obs*_ = 0.92).

### Tosufloxacin Tosylate

The total urinary excretion of TFLX (*Ur*, total of unchanged drug and metabolite) was reported to be 0.28 at a dose of 150 mg (TFLX TS) in the fasted state [46]. AUC was not affected by the co-administration of famotidine [62, 63]. As TFLX was little metabolized, *Fa* was assumed to be the same as *Ur* in the fasted state (*Fa*_*obs*_ = 0.28).

The food effect on the bioavailability of TFLX TS is positive (1.4-fold increase in AUC at 150 mg), suggesting that *Fa* in the fasted state is less than 0.71. This does not contradict the incomplete oral absorption of TFLX TS in the fasted state (cf. *Fa* in the fed state can be less than 1).

After oral administration of TFLX TS 150 mg tablets three times a day for three days in the fed state, the concentration of TFLX in the gallbladder bile was 5.80 to 12.52 μg/mL　[46]. Considering the gallbladder bile volume of about 50 mL, the TFLX amount in the gallbladder bile is less than 1% of a dose (0.29 to 0.63 mg). In rats, approximately 32% was excreted in bile after oral solution administration. Considering that TFLX would show high permeability (hence, *Fa* = 1 after solution administration), if the rat data is used for humans, *Fa* can be 0.41 (= 0.28/ (1–0.32)) in humans. However, biliary excretion is generally much less in humans than in rats [64]. The molecular weight cut-off value for biliary excretion is above 350 and 500 in rats and humans, respectively [64]. Based on MW of TFLX (MW = 404), the impact of biliary excretion on *Ur* can be minor in humans. Therefore, biliary excretion was neglected in the human *Fa* calculation.

### Levofloxacin (Free Form)

The absolute bioavailability (*F*) of LVFX FF is 1.0. AUC is dose-linear from 50 to 1000 mg [51]. AUC was not decreased by the co-administration of cimetidine. As LVFX was little metabolized, the observed *Fa* value (*Fa*_*obs*_) was assumed to be the same as *F* (*Fa*_*obs*_ = 1.0).

### Sitafloxacin (Free Form)

The *Ur* of a dose of 100 mg (STFX FF) was reported to be 0.73. AUC was not affected by the co-administration of ranitidine [65]. As STFX was little metabolized in humans and mainly excreted in urine, *Fa*_*obs*_ was assumed to be the same as *Ur* (*Fa*_*obs*_ = 0.73). In rats, the main route of elimination was glucuronidation followed by excretion in feces via the bile, while in monkeys it was excreted unchanged in the urine [65]. Therefore, like the case of TFLX, biliary excretion was neglected in the human *Fa* calculation for STFX.

## Results

### Physicochemical Properties of Model Drugs

The physicochemical properties of model drugs are shown in Table I. The first p*K*_*a*_ (p*K*_*a1*_) values are 5.6 to 6.2, corresponding to the carboxylic acid groups. The second p*K*_*a*_ (p*K*_*a2*_) values are 8.6 to 9.4, corresponding to the amine groups. The log *D*_*oct, pH6.5*_ value ranged from −1.38 (CPFX) to −0.14 (TFLX).

To avoid the change in pH_bulk_, *S*_*eq*_ was measured starting from the free form of each drug (CPFX FF anhydrate, TFLX FF 2H_2_O, GRNX FF 3H_2_O, and STFX FF 1.5H_2_O). The incubation time of 72 h is usually sufficient to achieve equilibrium [66, 67]. PPB was used for *S*_*eq*_ measurements because it was not possible to rigidly maintain the pH_bulk_ value of BCB for 72 h by the floating lid method. The final pH_bulk_ remained the same as the initial pH_bulk_ of 6.5.

The equilibrium solubilities in blank PPB-FaSSIF (without bile micelle) and PPB-FaSSIF (with bile micelle) were similar except for TFLX. The equilibrium solubility of LVFX was not measured as it has high intrinsic solubility (> 30 mg/mL) [51].

The residual solids after the solubility measurements were identified by PXRD and DSC as their corresponding free-form hydrates (CPFX FF hydrate Form III, TFLX FF 2H_2_O, GRNX FF 3H_2_O, and STFX FF 1.5H_2_O) (Figures S4 and S5, respectively).

### Estimation of Effective Intestinal Permeability

The *P*_*eff*_ value of CPFX was estimated to be 0.74 × 10^–4^ cm/s from the Caco-2 *P*_*app*_ data [42] by the *P*_*eff*_ equation (Eq. 4) (Table III). The *P*_*ep*_ values of GRNX, TFLX, LVFX, and STFX, were extrapolated from the Caco-2 *P*_*app*_ data of CPFX assuming a proportional relationship with *D*_*oct,pH6.5*_.

**Table III:** Permeability Data used for *Fa* Prediction

Drug	*P*_*ep*_ (× 10^–6^ cm/s)	*f*_*u*_	*P’*_*ep*_ (× 10^–4^ cm/s)	*D*_*mono*_ (× 10^–6^ cm^2^/s)	*D*_*eff*_ (× 10^–6^ cm^2^/s)	*P*_*UWL*_ (× 10^–4^ cm/s)	*P*_*eff*_ (× 10^–4^ cm/s)
CPFX	2.9	0.95	0.27	7.15	6.83	2.6	0.74
GRNX	26 ^a^	0.91	2.4	6.38	5.81	2.3	3.5
TFLX	50 ^a^	0.61	3.0	6.53	4.11	2.4	4.0
LVFX	19 ^a^	1.00^b^	1.9	6.87	6.87	2.5	3.2
STFX	13 ^a^	0.98	1.3	6.49	6.38	2.4	2.5

a Estimated from the *P*_*ep*_ value of CPFX by assuming being proportional to *D*_*oct, pH6.5*_

b LVFX was assumed not to bind bile micelles based on its low log *D*_*oct, pH6.5*_

### Dissolution Profiles

**Fig. 2:**
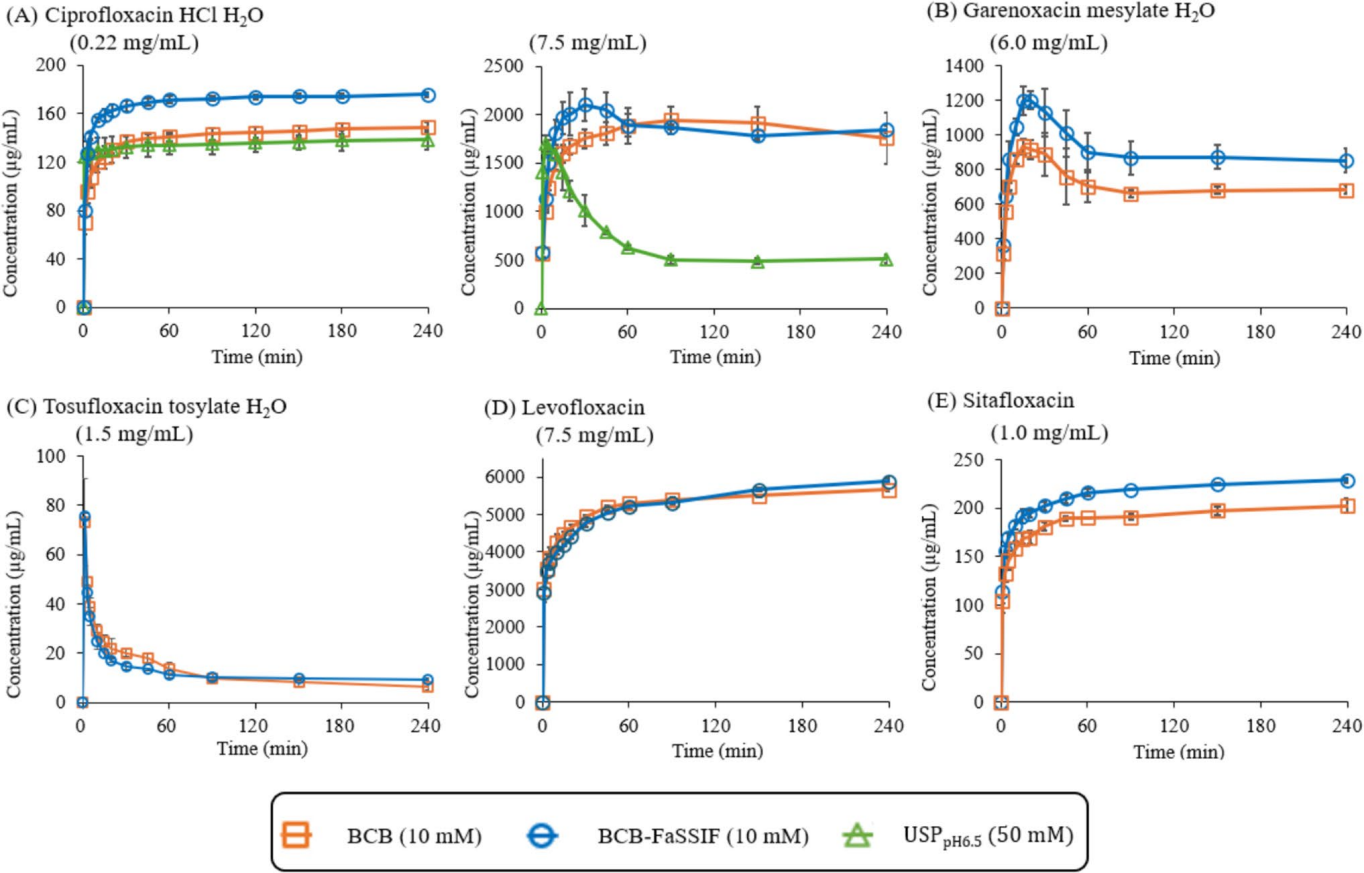
Dissolution profiles of model drugs

The dissolution profiles of the model drugs are shown in Fig. 2. The dissolution profile of TFLX TS in BCB was taken from the previous study [24]. Maximum *C*_*dissolv*_ was slightly higher in BCB-FaSSIF than in BCB in all cases, except for TFLX TS.

In the case of CPFX HCl, when using the compendial *Dose/FV* (200 mg/ 900 mL, 0.22 mg/mL), no supersaturation was observed in the dissolution test. The dissolution profile in USP_pH6.5_ was similar to that in BCB. *C*_*dissolv*_ approached the equilibrium solubility at pH 6.5 after 10 min. There was little or no change in pH_bulk_. *C*_*dissolv,AV*_ was about 0.13 to 0.17 mg/mL which is close to *S*_*eq,pH6.5*_. On the other hand, when using the clinical *Dose/FV* (750 mg/ 100 mL, 7.5 mg/mL), a supersaturation–precipitation profile was observed. The pH_bulk,4h_ value was markedly lower than pH 6.5. pH_bulk,4h_ in BCB and BCB-FaSSIF were lower than that in USP_pH6.5_. In BCB and BCB-FaSSIF, *C*_*dissolv*_ was maintained above 1.6 mg/mL for 4 h. In contrast, in USP_pH6.5_, rapid precipitation occurred after supersaturation. AUDC in USP_pH6.5_ was approximately three times lower than that of BCB and BCB-FaSSIF.

In the case of GRNX MS, a supersaturation-precipitation profile was observed in the dissolution test using the clinical *Dose/FV* (600 mg/ 100 mL, 6.0 mg/mL). The dissolution profile in BCB-FaSSIF was slightly higher than BCB. *C*_*dissolv*_ was about 0.7 mg/mL (BCB) and 0.9 mg/mL (BCB-FaSSIF) after 60 min. AUDC in BCB-FaSSIF was 1.3-fold higher than that in BCB. pH_bulk,4h_ was decreased from the initial pH (∆pH_bulk_ = −1.3) (Table IV).

**Table IV:** *Fa* prediction for CPFX HCl

*Dose/FV*^a^ (mg/mL)	Medium	AUDC ^*b*^ (mg /mL min)	*C*_*dissolv, AV*_ (mg/mL)	*pH*_*bulk,4h*_^*b*^	*Do *_*c*_	*Fa*_*pred*_^*f*^
7.50	BCB (10 mM)	382 ± 30	1.82	5.30 ± 0.00	3.2	0.49
	BCB-FaSSIF	392 ± 9	1.87	5.03 ± 0.04	3.1	0.50
	USP_pH6.5_	140 ± 4	0.67	5.66 ± 0.02	8.7	0.22
0.22	BCB (10 mM)	29.5 ± 0.9	0.14	6.46 ± 0.02	41	0.05
	BCB-FaSSIF	34.9 ± 0.5	0.17	6.44 ± 0.01	35	0.06
	USP_pH6.5_	27.9 ± 1.7	0.13	6.52 ± 0.01	43	0.05
4.0^d^	BCB (10 mM)^d^	300 ± 52.7	1.43	5.69 ± 0.04	4.0	0.41
4.0^d^	PPB (8 mM)^d^	618 ± 11	2.94	5.25 ± 0.01	2.0	0.66
-^e^	PPB-FaSSIF^e^	-	0.17^e^	-	36	0.06

a *Dose/FV* for the dissolution test

b Mean ± SD (N = 3)

c *Dose* = 750 mg and*V*_*SI*_ = 130 mL were used for *Do* calculation

d Adapted from the previous report [24]

e Equilibrium solubility in PPB-FaSSIF at pH 6.5 was used for *Fa* calculation

f Observed *Fa* (*Fa*_*obs*_) in the hypo-acidic gastric condition is 0.60 (at 750 mg dose)

In the case of TFLX TS, rapid precipitation was observed in the dissolution test using the clinical *Dose/FV* (150 mg/ 100 mL, 1.5 mg/mL). The dissolution profiles in BCB and BCB-FaSSIF were similar. *C*_*dissolv*_ was about 0.075 mg/mL at 1 min and dropped below 0.01 mg/mL after 60 min. pH_bulk,4h_ was decreased from the initial pH (∆pH_bulk_ = −0.9).

The dissolution profiles of LVFX FF in BCB and BCB FaSSIF were similar. pH_bulk,4h_ was slightly increased from the initial pH (∆pH_bulk_ = + 0.3). The final *C*_*dissolv*_ was about 5.9 mg/mL, which is below the theoretical maximum concentration (750 mg/ 100 mL, 7.5 mg/mL), indicating incomplete dissolution. The *C*_*dissolv*_ of STFX FF in BCB FaSSIF was slightly higher than that in BCB. *C*_*dissolv*_ approached the equilibrium solubility after 60 min. pH_bulk,4h_ was slightly increased from the initial pH (∆pH_bulk_ = + 0.3).

### Correlation Between Predicted and Observed Fa

The correlation between predicted and observed *Fa* was first investigated for CPFX HCl. The predicted *Fa* values (*Fa*_*pred*_) (clinical dose = 750 mg, *Fa*_*obs*_ = 0.60) are shown in Table IV. When using *S*_*eq,pH6.5*_ in PPB-FaSSIF (0.17 mg/mL) for *Do* calculation, *Fa* was tenfold underpredicted (*Fa*_*pred*_ = 0.06). When using compendial *Dose/FV* (0.22 mg/mL, 200 mg/ 900 mL) in the dissolution tests, *Fa* was also tenfold underpredicted in all cases of dissolution media. When using clinical *Dose/FV* (7.5 mg/mL, 750 mg/ 100 mL) and USP_pH6.5_, *Fa* was threefold underestimated (*Fa*_*pred*_ = 0.22). When using clinical *Dose/FV* and BCB or BCB-FaSSIF, *Fa*_*pred*_ was close to *Fa*_*obs*_. We previously reported *C*_*dissolv,AV*_ in 10 mM BCB and 8 mM PPB at *Dose/FV* = 4 mg/mL (1.43 and 2.94 mg/mL, respectively) [24]. Using these values, *Fa*_*pred*_ was 0.41 and 0.66, respectively.

The *Fa* predictability for the other fluoroquinolones was then investigated (Table V). In the case of GRNX MS, when using *S*_*eq*_ in PPB-FaSSIF (0.20 mg/mL) for *Do* calculation, *Fa* was threefold underpredicted (*Fa*_*obs*_ = 0.92,*Fa*_*pred*_ = 0.35). When using *C*_*dissolv,AV*_ in BCB and BCB-FaSSIF at clinical *Dose/FV* (6 mg/mL), *Fa*_*pred*_ was 0.79 (BCB) and 0.86 (BCB-FaSSIF), which are close to *Fa*_*obs*_. When using *C*_*dissolv,AV*_ in 10 mM BCB or 8 mM PPB at *Dose/FV* = 4 mg/mL we previously reported (0.50 and 0.74 mg/mL, respectively) [24], *Fa*_*pred*_ was 0.66 and 0.80, respectively, the latter is also close to *Fa*_*obs*_

**Table V:** *Fa* prediction for the Fluoroquinolones other than Ciprofloxacin

Drug/ Clinical dose ^a^ /*Fa*	*Dose/FV* (mg/mL) ^b^	Medium	AUDC (mg /mL min)	*C*_*dissolv, AV*_ *(mg/mL)*	*pH*_*bulk,4h*_	*Do*	*Fa*_*pred*_
GRNX MS	6.0	BCB (10 mM)	153 ± 10	0.73	5.26 ± 0.04	6.3	0.79
600 mg	6.0	BCB-FaSSIF	192 ± 16	0.91	5.21 ± 0.02	5.1	0.86
*Fa*_*obs*_ = 0.92	4.0^b^	BCB (10 mM)	105.9 ± 11.8^c^	0.50^c^	5.59 ± 0.01	9.2	0.66
	4.0^b^	PPB (8 mM)	154.7 ± 16.7^c^	0.74^c^	4.96 ± 0.01	6.2	0.80
	-	PPB-FaSSIF ^d^	- ^d^	0.20^d^	- ^d^	22.8	0.35
TFLX TS	1.5	BCB (10 mM)	3.03 ± 0.39	0.014	5.63 ± 0.02	80	0.13
150 mg	1.5	BCB-FaSSIF	2.63 ± 0.21	0.013	5.66 ± 0.00	92	0.12
*Fa*_*obs*_ = 0.28	1.5	PPB (8 mM)	7.46 ± 1.31 ^b^	0.036^b^	3.77 ± 0.01	32	0.30
	-	PPB-FaSSIF ^c^	- ^d^	0.0041 ^d^	- ^d^	281	0.04
	0.17^e^	PPB (25 mM) ^e^	1.55 ^e^	0.0070 ^e^	-	165	0.07
LVFX FF	7.5	BCB (10 mM)	1105 ± 5	5.3	6.81 ± 0.01	1.1	1.0
750 mg	7.5	BCB-FaSSIF	1107 ± 6	5.3	6.84 ± 0.01	1.1	1.0
*Fa*_*obs*_ = 1.0	-	-	-	> 30	-	< 1	1.0
STFX FF	1.0	BCB (10 mM)	39.7 ± 0.09	0.19	6.76 ± 0.01	4.1	0.82
100 mg	1.0	BCB-FaSSIF	45.2 ± 0.34	0.22	6.78 ± 0.01	3.6	0.86
*Fa*_*obs*_ = 0.73	-	PPB-FaSSIF ^c^	- ^d^	0.181 ^d^	- ^d^	4.2	0.81

a Clinical dose for observed *Fa* (*Fa*_*obs*_) in the hypo-acidic gastric condition

b *Dose/FV* for dissolution test

c Adapted from the previous report [24]

d Equilibrium solubility in PPB-FaSSIF at pH 6.5 was used for *Fa* calculation

e From the result of the Japanese compendial dissolution test (pH 6.8, 25 mM PPB, *Dose/FV* = 0.17 mg/mL (150 mg/ 900 mL, paddle rotation speed: 50 rpm)) [46]

In the case of TFLX TS, when using *S*_*eq*_ in PPB-FaSSIF (4.1 μg/mL) for *Do* calculation, *Fa* was sevenfold underpredicted (*Fa*_*obs*_ = 0.28, *Fa*_*pred*_ = 0.04). When using the result of a compendial dissolution test reported in the literature (pH 6.8 PPB (25 mM) at *Dose/FV* = 0.17 mg/mL, *C*_*dissolv,AV*_ = 7 μg/mL [46]), *Fa* was fourfold underpredicted (*Fa*_*pred*_ = 0.07). When using clinical *Dose/FV* (1.5 mg/mL, 150 mg/ 100 mL) and BCB, *Fa* predictability was improved, however, still twofold underpredicted (*Fa*_*pred*_ = 0.13). Similar results were obtained with BCB-FaSSIF. When using *C*_*dissolv,AV*_ in 8 mM PPB we previously reported (*C*_*dissolv,AV*_ = 36 μg/mL, pH_bulk,4h_ = 3.8) [24], *Fa*_*pred*_ was 0.30, which is close to *Fa*_*obs*_*.*

The *Fa* value of LVFX FF was predicted to be 1.0 from *C*_*dissolv,AV*_ in both BCB and BCB-FaSSIF, respectively, matching the clinical *Fa*. The *Fa* value of STFX FF was predicted to be 0.82 (BCB) and 0.86 (BCB-FaSSIF), close to *Fa*_*obs*_ = 0.73.

**Fig. 3:**
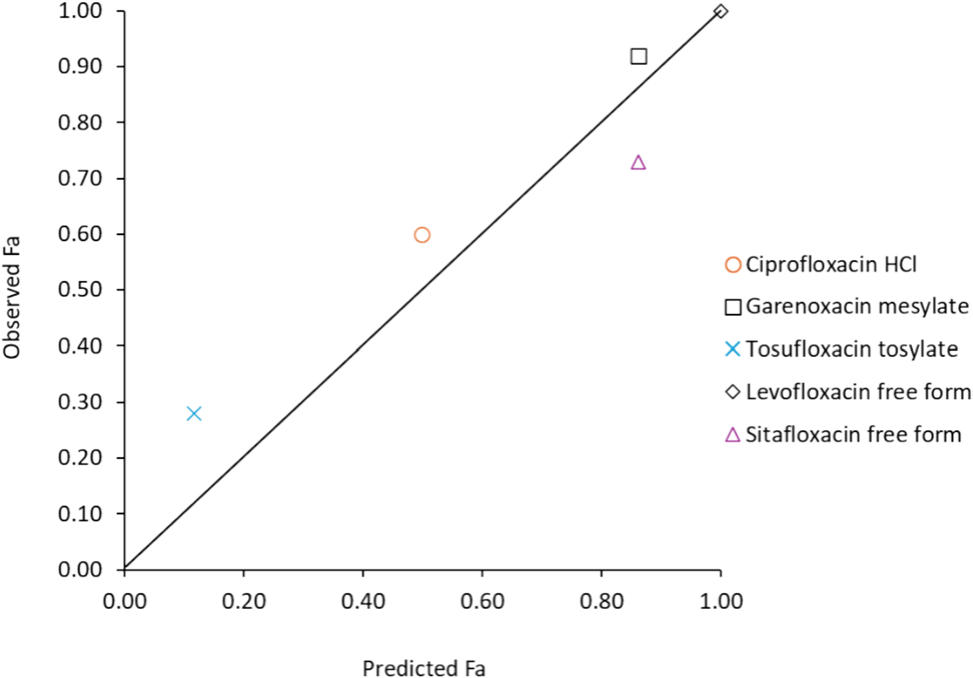
Predicted vs. observed *Fa* based on the dissolution profiles in BCB-FaSSIF at clinical *Dose/FV.*

*Fa*_*obs*_ and *Fa*_*pred*_ based on the dissolution profiles in BCB-FaSSIF at clinical *Dose/FV* are plotted in Fig. 3. The coefficient of determination (*r*^*2*^) was 0.97. The average absolute fold error was 1.4.

## Discussion

This study investigated the correlation between the dissolution profiles of salt-form drugs at clinical *Dose/FV* and clinical *Fa*. Previously, it was qualitatively suggested that the use of clinical *Dose/FV* would be important for *Fa* prediction based on biorelevant dissolution tests because it affects pH_bulk_ for various salt-form drugs [24]. In this study, the *Fa* predictability was quantitatively evaluated using fluoroquinolones as model drugs.

### Physicochemical Properties of Model Drugs

Based on the p*K*_*a*_ values, all model drugs mainly exist in a zwitterionic form at pH 6.5 in a solution. All model drugs are hydrophilic at pH 6.5 (log *D*_*oct,pH6.5*_ < 0). However, except for LVFX, these drugs showed low solubility at pH 6.5. Zwitterionic fluoroquinolones exhibit a U-shaped pH solubility profile with a solubility minimum at the isoelectric point (= (p*K*_*a1*_ + p*K*_*a2*_)/ 2) [44]. *S*_*eq*_ was little increased by bile micelles in good agreement with their low lipophilicity, except for TFLX which has the highest log *D*_*oct,pH6.5*_*.* Based on *S*_*eq*_, the dissolution test was suggested to be under a non-sink condition (*Dose/FV* > *S*_*eq*_) except for LVFX.

### Estimation of Effective Intestinal Permeability

The estimated *P*_*eff*_ value of CPFX in humans (0.74 × 10^–4^ cm/s) is lower than that of metoprolol [68]. Therefore, CPFX is categorized as a low-permeability drug according to the biopharmaceutics classification system [69].

In the literature, the Caco-2 *P*_*app*_ values of CPFX were reported to be low to moderate (0.42 to 3.3 × 10^–6^ cm/s, mostly 2.5 to 3.3 × 10^–6^ cm/s) [42]. In the present study, *P*_*app*_ = 2.9 × 10^–6^ cm/s was used as a typical Caco-2 *P*_*app*_ value [70] for human *P*_*eff*_ estimation. In the rat intestinal perfusion studies, the *P*_*eff*_ values of CPFX in rats were reported to be 8 to 17 × 10^–6^ cm/s [71], which corresponds to *P*_*eff*_ = 0.48 to 1.0 × 10^–4^ cm/s in humans (*PE* = 1 and*VE* = 5 for rats [72]), in good agreement with the estimation from Caco-2 *P*_*app*_. In the parallel artificial membrane permeation assay, the permeation of CPFX was low to moderate [42, 73]. The log*D*_*oct,pH6.5*_ value of CPFX (−1.38) also supports its low passive permeability. Taken together, the human *P*_*eff*_ value for CPFX was considered reasonable. The *P*_*ep*_ value of zwitterions would be little affected by pH between p*K*_*a1*_ and p*K*_*a2*_ [42]. In addition, the microclimate pH adjacent to the epithelial membrane *in vivo* is robustly maintained at about pH 6.0 to 6.5 [74], not affected by pH_bulk_. The *P*_*eff*_ values of CPFX, LVFX, and STFX were suggested to be limited by *P*_*ep*_ (*P*_*ep*_*’* < *P*_*UWL*_), whereas those of GRNX and TFLX were slightly above the borderline between *P*_*ep*_ and UWL limited cases. Therefore, the particle drifting effect in the UWL was neglected in the *P*_*eff*_ calculation [75, 76].

### Dissolution Profiles

In the case of the salt-form drugs (CPFX HCl, GRNX MS, and TFLX TS), at the clinical *Dose/FV*, pH_bulk,4h_ in BCB and BCB-FaSSIF was decreased below its first p*K*_*a*_ (p*K*_*a1*_), due to its insufficient buffer capacity. These results are in good agreement with our previous study [24]. *S*_*eq*_ in the pH < p*K*_*a1*_ range becomes higher than that at pH 6.5, resulting in less supersaturation, less precipitation, and consequently higher *C*_*dissolv,AV*_, compared to the cases when pH_bulk_ is maintained at pH 6.5 by sufficient buffer capacity (β > > *Dose/FV* (mol/L)). In the case of CPFX HCl, at compendial *Dose/FV* (0.22 mg/mL, 0.6 mmol/L)*,* the buffer capacity of 10 mM BCB (4.4 mmol/pH unit) was sufficient so that the pH_bulk_ remained at pH 6.5 and *C*_*dissolv,AV*_ became close to *S*_*eq*_ at pH 6.5.

In the case of STFX FF, *C*_*dissolv,AV*_ was similar to* S*_*eq*_ at pH 6.5. pH_bulk_ was slightly increased due to neutralization by the drug (the isoelectric point is about pH 7.4). However, pH_bulk_ was between p*K*_*a1*_ and p*K*_*a2*_. Therefore, *C*_*dissolv,AV*_ was little affected by the pH change. Similarly, pH_bulk_ was slightly increased in the case of LVFX FF. From the equilibrium solubility of LVFX, complete dissolution was expected. However, the dissolution of LVFX from the tablet formulation was incomplete. The reason for the incomplete dissolution was not clear. In the case of TFLX TS, even though *S*_*eq,pH6.5*_ was about twofold increased by bile micelles, little difference was observed in the dissolution profiles between BCB and BCB-FaSSIF. The reason is under investigation.

### Fa Predictability

In this study, the *Fa* values were predicted by the GUT framework [41]. Its *Fa* predictability has been validated by systematic studies for structurally diverse poorly soluble neutral, free weak acidic, and free weak base drugs, with an absolute average fold error (AAFE) of 1.5 [5, 6]. In this study, the *Fa* values of free-form zwitterionic fluoroquinolones (LVFX FF and STFX FF) were adequately predicted, confirming that the dissolution test conditions and the GUT framework were suitable for zwitterionic fluoroquinolones.

In the case of CPFX HCl, when using clinical *Dose/FV* (7.5 mg/mL) in the dissolution test, the clinical *Fa* value was adequately predicted by both BCB media. *Do* was calculated to be above 1 (*Do* = 3.1), suggesting that the oral absorption of CPFX HCl would become less than dose proportional. This is in good agreement with the clinical data that AUC shows a slightly less than dose-proportional increase from 50 to 750 mg [59–61]. When using USP_pH6.5_, *Fa* was about threefold underestimated, suggesting that *in vivo* precipitation was overestimated in this condition. When using compendial *Dose/FV* (0.22 mg/mL), *Fa* was underestimated by one order of magnitude. These results suggested that the use of clinical *Dose/FV* would be important for the dissolution test using biorelevant BCB media.

The *Fa* predictability of two other fluoroquinolone salts was then investigated. Similar to CPFX HCl, in both cases of GRNX MS and TFLX TS, *Fa* was significantly underestimated when using* S*_*eq*_ at pH 6.5. When using *C*_*dissolv,AV*_ in BCB or BCB-FaSSIF at clinical *Dose/FV*, *Fa* was adequately predicted for GRNX MS. In the case of TFLX TS, the *Fa* predictability was also improved, however, *Fa* was still twofold underpredicted. TFLX TS has the highest lipophilicity and was predicted to have the highest *P*_*eff*_ among the model drugs. Drug permeation across the intestinal wall lowers *C*_*dissolv*_ and decreases the precipitation rate in the small intestine [77]. Therefore, precipitation might have been overestimated by a dissolution test in the absence of permeation clearance for high-permeability drugs. In addition, the TFLX TS tablet contains L-aspartic acid as an acidifier [46]. This might also have complicated the *Fa* prediction by a dissolution test.

### Phosphate Buffer as a Surrogate of Bicarbonate Buffer

The *Fa* value of TFLX from the TFLX TS tablet was most appropriately predicted using 8 mM PPB for the dissolution test at clinical *Dose/FV* [24], suggesting that it may be possible to find a surrogate buffer optimized for each case of drug and formulation focusing on either pH_surface_ or pH_bulk_. In that condition, pH_bulk,4h_ became 3.77, which is much lower than the case of 10 mM BCB (pH_bulk,4h_ = 5.63) [24]. As mentioned in the introduction, the pH maintenance efficacy of PPB against acidification at pH 6.5 is lower than that of BCB, even when β is set to be the same [24]. In the case of the TFLX TS tablet, this may have coincidently canceled out an overestimation of precipitation due to the lack of permeation clearance in dissolution testing.

### Practical use of Bicarbonate-Based Biorelevant Media in Drug Discovery and Development

Theoretically, it is impossible to simultaneously adjust both pH_surface_ and pH_bulk_ between BCB and the other buffers [22]. For example, the concentration of PPB must be set much lower than BCB to adjust pH_surface_ because BCB has a slower pH neutralization rate due to the slow hydration/dehydration rates of CO_2_ [18, 25, 78]. On the other hand, to adjust pH_bulk_, the concentration of PPB must be set higher than BCB because pH_bulk_ maintenance efficacy of PPB for acidification is lower than that of BCB [22]. An optimal *in vivo* predictive surrogate buffer would be different for each counter ion of salt and excipient [25, 79]. Therefore, it may be difficult to find a surrogate buffer for screening salt forms and formulations.

In this study, BCB-FaSSIF was employed as a practical biorelevant simulated intestinal fluid for a dissolution test in drug discovery and development [55]. The floating lid method is simple, easy, and robust. The floating lid has been successfully applied to a small-scale dissolution apparatus for use in drug discovery such as a mini-vessel [80] and μDISS [81, 82]. It has also been applied to a pH-shift dissolution test [83] and the flow-through dissolution test [84]. To improve the *in vivo* predictability, it would be preferable to use BCB for salt form and formulation screenings in drug discovery and development. Subsequently, for quality control, an alternative buffer may be developed that gives an *in vitro*–*in vivo* correlation similar to BCB. However, as the floating lid method is robust and reproducible, it may also be usable for quality control.

### Suggestion for Physiologically Based Biopharmaceutics Modeling

The results of this study suggest that the effect of a salt-form drug on pH_bulk_ in the small intestine should be considered in physiologically based biopharmaceutics modeling (PBBM) [24]. In the previous PBBM studies of CPFX HCl [85–88], the middle-out approach (parameter fitting to clinical PK profiles) was used to recover clinical PK profiles [89], presumably because the prediction from *in vitro* data had failed. In those studies, the *S*_*eq*_ of CPFX was deliberately set high (e.g., > 30 mg/mL at pH 7.0) and a high *P*_*eff*_ value was back estimated from the clinical PK data by parameter fitting (e.g., *P*_*eff*_ > 2 × 10^–4^ cm/s). However, these *S*_*eq*_ and *P*_*eff*_ values contradict the *in vitro* data of CPFX [71]. Parameter fitting can mask any errors in PBBM [90]. In the previous PBBM studies, the intestinal pH_bulk_ might have been assumed to be not affected by the administration of a drug [85–88]. However, this assumption is at least questionable. Previously, the intestinal pH_bulk_ was reported to be decreased after the administration of 800 mg ibuprofen in humans [91, 92]. The large interindividual variability of the intestinal pH_bulk_ suggests that it is not tightly regulated [93, 94]. Therefore, it would rather be realistic to assume that the small intestinal pH_bulk_ can be affected by an administered drug.

It should be noted that, by definition, the *equilibrium* solubility at an *equilibrium* (final) pH (*S*_*eq*_) became the same regardless of the starting material being a free-form or salt-form in the pH-controlled region (below/ above pH_max_ for acids/ bases, respectively) [95]. In this pH region, the equilibrium maker (= residual solid) is free form. In a *S*_*eq*_ measurement, when the buffer capacity is not enough to maintain pH_bulk_, it is altered by adding an excess amount of an ionizable drug. This is especially often observed when using a salt-form drug for a *S*_*eq*_ measurement. In the pH-controlled region, as the salt form transforms to a free form, the counter acid or base is released, resulting in a pH change [96, 97]. The drug concentration in the solution measured in such a way is neither the *S*_*eq*_ at the initial pH nor the salt solubility (salt solubility is defined differently from *S*_*eq*_ [95–97]). The final pH_bulk_ and the residual solid form should always be checked for *S*_*eq*_ measurements [66, 67]. As mentioned in the introduction, it is difficult to predict the dissolution/supersaturation/precipitation profile of a salt-form drug based on its intrinsic physicochemical properties. At least, the solubility product (*K*_*sp*_) is required to calculate the dissolution rate of a salt-form solid. In addition, a mechanistic or semi-mechanistic model for the nucleation process is required to calculate supersaturation/ precipitation processes [32, 36].

### Limitations of this Study

The clinical *Fa* values of TFLX TS and STFX FF were calculated based on urinary excretion data assuming little drug metabolism and biliary excretion because the pharmacokinetic data after intravenous administration was not available. However, the other elimination routes such as biliary excretion may affect *Ur*. Therefore, those *Fa* values would be regarded as minimum estimates for these drugs. As discussed for TFLX, in the case of high-permeability drugs, permeation clearance reduces the drug concentration in the intestinal fluid, resulting in reduced drug precipitation. Therefore, the prediction approach of this study applies to low to moderate-permeability drugs, but not high-permeability drugs. In this study, the tablet disintegration process was not considered because the crushed tablet was used to adjust the *Dose*/*FV* ratios. The immediate release formulations with rapid disintegration were used in this study. In this case, the disintegration process may affect *C*_*max*_ but would have little effect on *Fa*.

In conclusion, for salt-form drugs, *C*_*dissolv,AV*_ in the dissolution test using a biorelevant BCB medium at clinical *Dose/FV* was significantly higher than *S*_*eq*_ at oH 6.5 due to the pH_bulk_ reduction by the added drug. The clinical *Fa* values of CPFX HCl, GRNX MS, STFX FF, and LVFX FF were adequately predicted (AAFE < 1.2) from the dissolution profiles, whereas that of TFLX TS was twofold underpredicted. The use of clinical *Dose/FV* and biorelevant dissolution media was suggested to be important to improve the correlation between the dissolution profiles and clinical *Fa* for salt-form drugs. For high-permeability drugs, the effect of permeation clearance on drug precipitation should be considered for *Fa* prediction.

## Supplementary Information

Below is the link to the electronic supplementary material.

ESM 1 Supplementary file1 (DOCX 1.00 MB)

## Data Availability

The datasets generated during and/or analyzed during the current study are available from the corresponding author on request.
