# Supplementary material for: Correlation Between Dissolution Profiles of Salt-Form Drugs in Biorelevant Bicarbonate Buffer and Oral Drug Absorption: Importance of Dose/ Fluid Volume Ratio
Source: Pharm Res. 2025 Apr 4;42(4):623–37. doi: 10.1007/s11095-025-03854-y (PMC12055928; doi:10.1007/s11095-025-03854-y)
Supplement: Supplementary file 1 — Supplementary file1 (DOCX 1.00 MB) [file 11095_2025_3854_MOESM1_ESM.docx]

Supplemental information

Correlation between dissolution profiles of salt-form drugs in biorelevant bicarbonate buffer and oral drug absorption: importance of dose/ fluid volume ratio

Yuki Tarumi, Yuji Higashiguchi, Kiyohiko Sugano^*^

Molecular Pharmaceutics Lab., College of Pharmaceutical Sciences, Ritsumeikan University, 1-1-1, Noji-higashi, Kusatsu, Shiga 525-8577, Japan

* Corresponding author. Tel.: +81-77-561-2773; E-mail address: suganok@fc.ritsumei.ac.jp (K. Sugano).


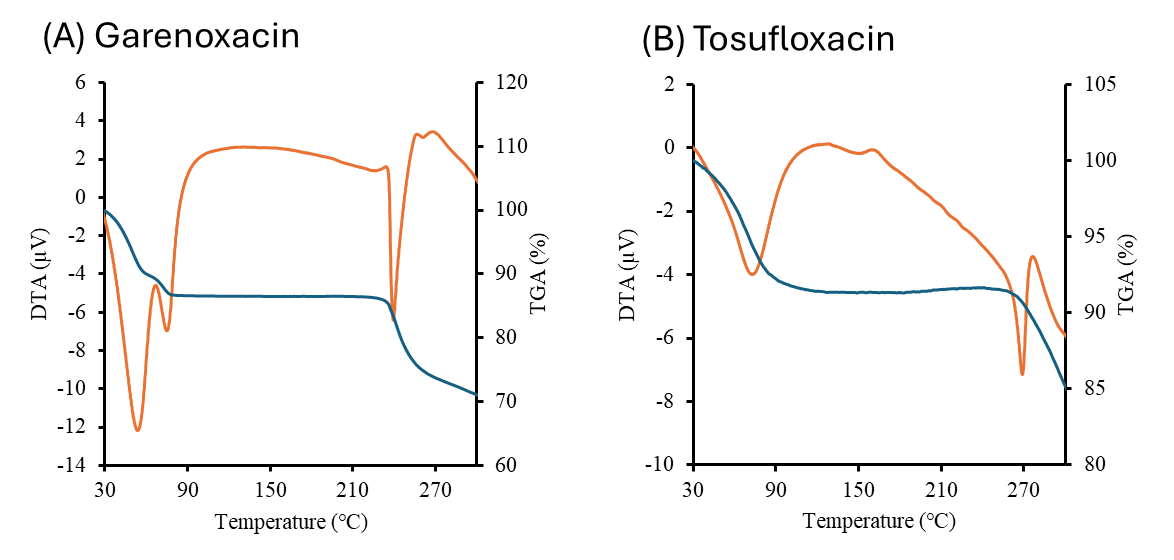


Figure S1 Thermogravimetric analysis (TGA) data of the reference materials of garenoxacin free form (A) and tosufloxacin free form (B). In the TGA measurement (DTG-60AH, Shimadzu Corporation Kyoto, Japan), samples were placed in an aluminum pan (non-sealed) and measured under nitrogen gas at 10℃/min.


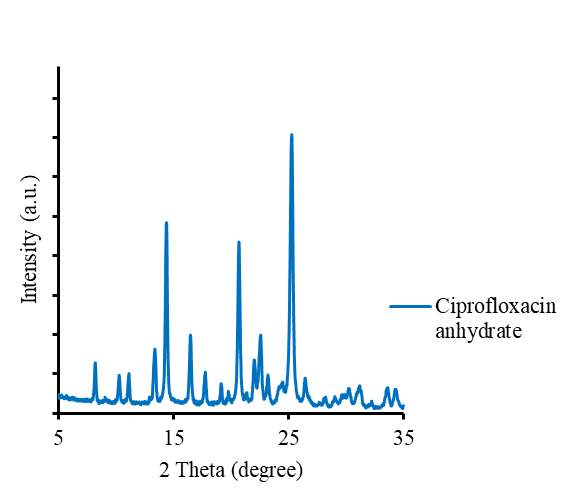


Figure S2 PXRD data of ciprofloxacin free form anhydrate

Table S1 The wavelength and standard curves for log*D* measurement (log *D*_oct,pH6.5_)

| Drug | UV detection wavelength | Concentration range (mM) | *r^2^* |
| --- | --- | --- | --- |
| Garenoxacin | 325 | 0.0016 - 0.050 | 0.9997 |
| Sitafloxacin | 350 | 0.0016 - 0.050 | 1.0000 |

Table S2 The final pH, wavelength, and standard curves for solubility measurement

| Drug | Media | Final pH^a^ | UV wavelength | Concentration range (µg/mL) | *r^2^* |
| --- | --- | --- | --- | --- | --- |
| Ciprofloxacin | blank PPB-FaSSIF | 6.50 ± 0.00 | 316 | 3.91 - 62.50 | 0.9999 |
|  | PPB-FaSSIF | 6.51 ± 0.00 |  |  | 0.9995 |
| Garenoxacin | blank PPB-FaSSIF | 6.50 ± 0.00 | 325 | 1.82 – 29.05 | 0.9999 |
|  | PPB-FaSSIF | 6.50 ± 0.00 |  |  | 1.0000 |
| Tosufloxacin | blank PPB-FaSSIF | 6.49 ± 0.01 | 365 | 0.74 – 23.64 | 0.9998 |
|  | PPB-FaSSIF | 6.50 ± 0.00 |  |  | 0.9981 |
| Sitafloxacin | blank PPB-FaSSIF | 6.49 ± 0.00 | 350 | 1.75 - 28.06 | 1.0000 |
|  | PPB-FaSSIF | 6.50 ± 0.00 |  |  | 1.0000 |

a Mean ± S.D., N = 3.

Table S3 Wavelength and standard curves for dissolution test

| Drug | Media | UV wavelength | Concentration range (µg/mL) | *r^2^* |
| --- | --- | --- | --- | --- |
| Ciprofloxacin | BCB | 310 | 0.670 - 42.85 | 1.0000 |
| (375 mg/50 mL) | BCB-FaSSIF |  |  | 0.9999 |
|  | USP-2 |  |  | 1.0000 |
| (200 mg/900 mL) | BCB |  |  | 1.0000 |
|  | BCB-FaSSIF |  |  | 0.9998 |
|  | USP-2 |  |  | 1.0000 |
| Garenoxacin | BCB | 325 | 0.454 - 29.05 | 0.9997 |
|  | BCB-FaSSIF |  |  | 0.9998 |
| Tosufloxacin | BCB | 365 | 0.408 - 26.13 | 1.0000 |
|  | BCB-FaSSIF |  |  | 1.0000 |
| Levofloxacin | BCB | 335 | 0.798 - 51.07 | 1.0000 |
|  | BCB-FaSSIF |  |  | 1.0000 |
| Sitafloxacin | BCB | 350 | 0.438 - 28.06 | 1.0000 |
|  | BCB-FaSSIF |  |  | 1.0000 |


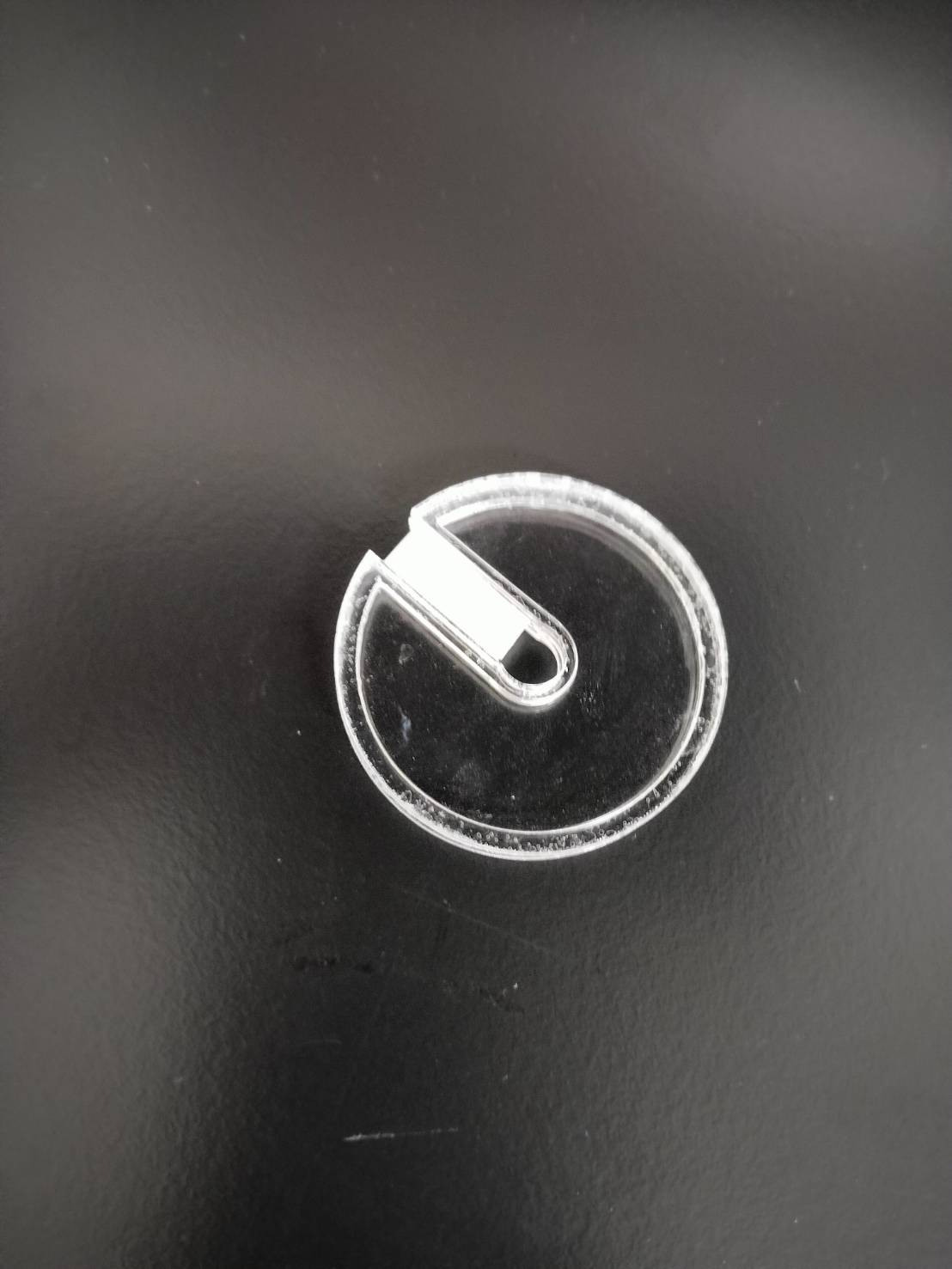


Figure S3 Floating lid for the mini vessel. The floating lid was made of 2 mm thick acrylic plate. Diameter: 49.5 mm, diameter of central hole: 8.0 mm, slit width: 7.4 mm. The floating lid was made of two plates and a central frame to form a cavity. Styrofoam was used to fill the slit.


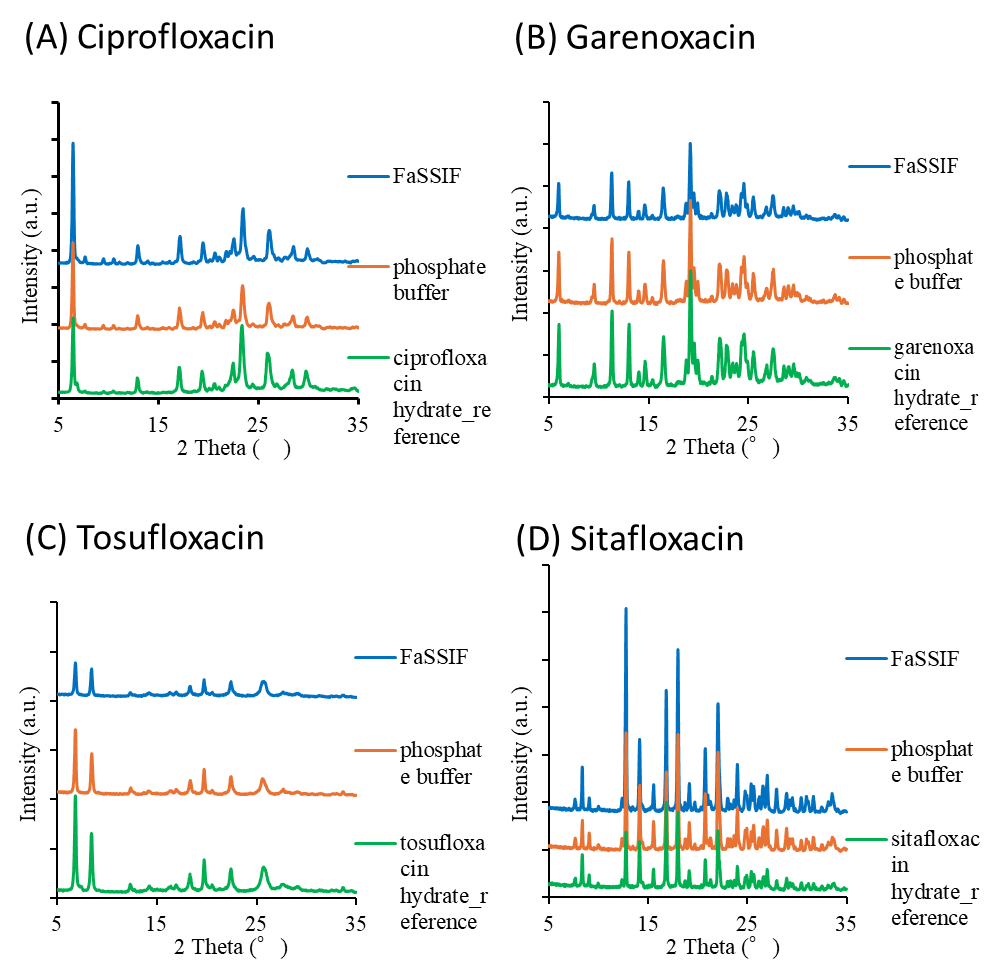


Figure S4 PXRD patterns of the residual solid in the solubility measurement. (A) Ciprofloxacin, (B) garenoxacin, (C) tosufloxacin, and (D) sitafloxacin.


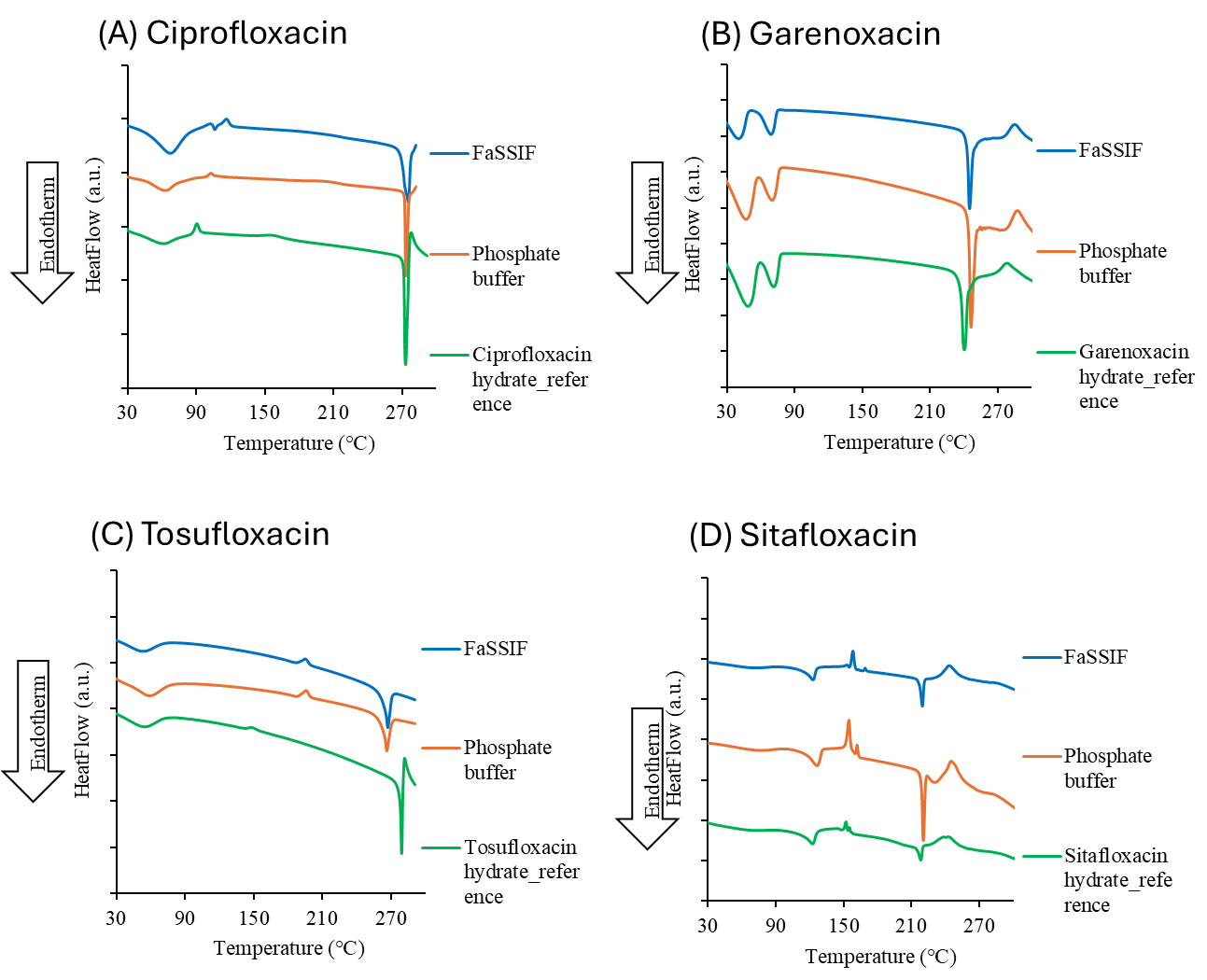


Figure S5 DSC curves of the residual solid in the solubility measurement. (A) Ciprofloxacin, (B) garenoxacin, (C) tosufloxacin, and (D) sitafloxacin.
